# Daily Exercise Patterns and Their Differences between Parkinson's Disease Patients with and without Postural Instability

**DOI:** 10.1155/2022/3191598

**Published:** 2022-05-19

**Authors:** Joomee Song, Jun Kyu Mun, Jong Hyeon Ahn, Jinyoung Youn, Inyoung Choi, Jin Whan Cho

**Affiliations:** ^1^Department of Neurology, Samsung Medical Center, Sungkyunkwan University School of Medicine, Seoul, Republic of Korea; ^2^Neuroscience Center, Samsung Medical Center, Seoul, Republic of Korea

## Abstract

**Background:**

Due to the clinical impact of exercise in patients with Parkinson's disease (PD), management should include personalized and effective exercises according to patient's PD stage. We investigated the detailed exercise behaviors of patients with mild to advanced PD and compared their patterns between PD with and without postural instability (PI).

**Methods:**

We enrolled PD patients from September to December 2019. Clinical data on parkinsonism, exercise behaviors, and Physical Activity Scale of the Elderly (PASE) scores were collected and compared between mild PD without PI (Hoehn–Yahr (HY) stages 1 and 2) and advanced PD with PI (HY stages 3 and 4).

**Results:**

In total, 263 PD patients were recruited. The mean exercise frequency was 4.7 ± 2.1 times/week, and the average duration was 7.8 ± 6.7 hours/week. The most common exercise was an aerobic exercise (71.9%) of mild-to-moderate intensity, with active walking being the most common (49.0%). The mild PD patients demonstrated a higher duration and intensity of exercise and more physical activity than the advanced PD patients. However, the frequency of exercise was not significantly different between the two groups. The PASE score was significantly higher in mild PD patients than in advanced PD patients (*p* < 0.001).

**Conclusion:**

PD patients focused mostly on aerobic exercises, especially active walking. With the disease progression, the amount and intensity of exercise decreased while frequency remained. Higher intensity of exercise is needed in the mild PD group, while the advanced PD group requires the increment of duration for each exercise session.

## 1. Introduction

Parkinson's disease (PD) is a neurodegenerative disorder, and its treatment has traditionally focused on symptomatic management along with dopamine replacement therapy [1]. Unlike pharmacological treatment, exercise improves various motor and nonmotor symptoms in PD without worsening dyskinesia or producing significant side effects [2–5]. In addition, previous studies have revealed a possible disease-modifying effect from exercise in PD patients [4, 6]. Despite some guidelines for exercise in PD patients [7, 8], much controversy remains regarding the detailed protocols for exercise in PD patients. While standardized aerobic exercises were the primary method in most of the previous studies, several studies have approached various kinds of exercises such as land and water-based exercise [9] or mat Pilates [10] and showed positive effects on motor function and quality of life. High-intensity aerobic workouts with a lower extremity cycle ergometer were feasible in PD patients and improved motor symptoms [11]. Still, the exercise is a tremendously complicated behavior depending on various environmental and personal factors. The exercise recommendations should be based on the patients' condition, such as the living area, any comorbidities, and the patient's confidence for exercise.

More importantly, as PD progresses, exercise recommendations should also evolve to adapt to the symptom severity of the patients. Among the motor symptoms of PD, postural instability (PI), which develops as the disease progresses, has been reported to contribute significantly to both physical inactivity and decreased ADL than other motor symptoms [12–14]. Therefore, it is necessary to understand the exercise behaviors of patients in various stages of PD because these data would be the first step to personalize exercise programs for specific stages of PD and to establish guidelines for effective exercises as part of a treatment plan for PD patients. As part of the exercise behaviors, the exercise type, frequency, and intensity are the most basic parameters, but there is a paucity of knowledge on the best exercise patterns to recommend depending on PD severity.

In this study, we investigated the exercise patterns which include exercise type, frequency, and intensity of PD patients and compared it between PD without PI and PD with PI patients. While investigating the exercise patterns, we also investigated physical activity levels of the patients including intensity of work or household chores. The aim was to identify a baseline for exercise recommendations in PD patients and also to establish necessary changes for PD progression, which could serve as a reference for personalized exercise recommendations for PD patients in the future.

## 2. Methods

### 2.1. Participants and Clinical Assessments

We enrolled PD patients who were able to walk independently (Hoehn–Yahr (HY) stage ≤4) [15] from September to December 2019 at the Movement Disorders Clinic of Samsung Medical Center in Seoul, Korea. PD was diagnosed according to the United Kingdom Parkinson's Disease Society Brain Bank criteria [16]. We excluded patients with Parkinson-plus syndromes, including multiple systemic atrophy, progressive supranuclear palsy, and corticobasal syndrome, vascular parkinsonism, drug-induced parkinsonism, or normal pressure hydrocephalus; structural brain lesions, including stroke or tumor; cardiopulmonary, musculoskeletal problems, or other neurological conditions (e.g., myelopathy, known neuropathy, and chronic vestibular dysfunction) that preclude any exercise; severe cognitive impairment (4 ≤ Global Deterioration Scale (GDS) score) [17, 18]; and psychiatric diseases requiring medical treatment, including major depressive disorder, bipolar and related disorders, and schizoaffective disorders diagnosed according to DSM-V criteria [19].

Parkinsonian motor symptoms were evaluated using the unified Parkinson's disease rating scale (UPDRS) part 3 and the Hoehn–Yahr (HY) stage in the medication “on” state [15]. The dose of dopaminergic medications was checked using the levodopa equivalent daily dose (LEDD) based on previous literature [20]. We divided all recruited patients into either the mild PD group (HY stages 1-2) who did not have PI or the advanced PD group (HY stages 3-4) who have PI.

### 2.2. Exercise Behavior and Physical Activity Level Evaluation

We interviewed all the enrolled participants, and they reported the types, duration (hours/session), and frequency (times/week) of recently completed exercises. When they reported multiple exercises, the most frequently preformed exercise was designated as the primary exercise, followed by any secondary and tertiary exercises. The primary exercises were categorized as follows based on the physiology of exercise: aerobic exercise/resistance exercise/stretching [21]. We classified exercises into categories corresponding to their major components. When the major component was obscure, we followed previous studies' classifications which utilized the exercises. Also, we classified the exercises according to home-based (indoor or outdoor)/at sports facilities and solo/group exercise. The types of exercises corresponding to each category are summarized in Supplementary Table 1. The intensity of the primary exercises done by patients was measured using metabolic equivalents (METs), and the exercises were classified as mild (METs ≤ 3), moderate (4 ≤ METs ≤ 6), or high-intensity (7 ≤ METs) exercise [22].

The amount of physical activity undertaken by the included PD patients was evaluated using the Korean version of the Physical Activity Scale of the Elderly (PASE), a validated, self-reported questionnaire that assesses the level of activity over the prior week [23, 24]. Higher PASE scores indicate more physical activity, with scores ranging from 0 to 365 in the validated sample. The final score is calculated by adding up three sections: leisure exercise, work/volunteering, and household chores.

Finally, we classified patients into either the mild PD group (HY stages 1-2) or the advanced PD group (HY stages 3-4) based on the presence of postural instability and compared the exercise patterns between these two groups.

### 2.3. Statistical Analysis

All data are presented as the mean ± standard deviation. The demographic and clinical data of the mild PD and moderate-to-severe PD groups were compared using Student's *t*-tests or Mann–Whitney *U*-tests for continuous variables and Pearson's *χ*^2^ or Fisher's exact tests for categorical variables. A *p* value <0.05 was considered significant. Statistical analyses were performed using a commercially available software package (SPSS, Version 25.0., IBM Corp., Armonk, NY, USA).

## 3. Results

### 3.1. Demographic and Clinical Characteristics of Recruited Participants

**Figure 1:**
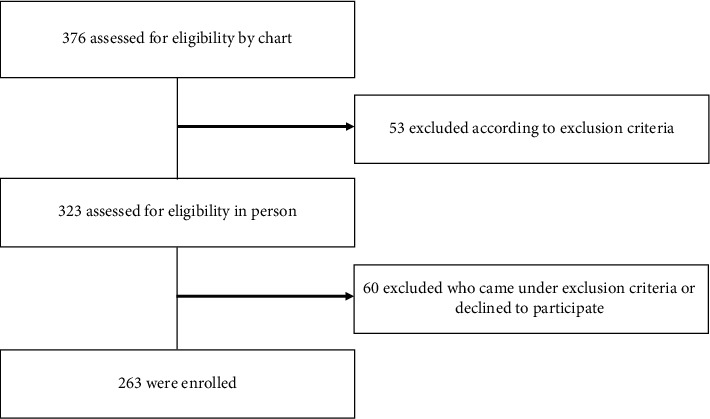
Flowchart to describe the enrolled study population.

We screened 323 eligible patients based on our movement clinic records, and 309 were assessed for eligibility in person (Figure 1). In total of 263 PD patients finally enrolled, 210 (79.8%) participants were classified into the mild PD group, while 53 (20.2%) were placed in the advanced PD group. Their mean age was 67.7 ± 29.2 years, and the average disease duration was 8.3 ± 5.2 years. All the demographics, clinical data, and exercise characteristics are given in Table 1.

### 3.2. Exercise Patterns of PD Patients

The mean exercise frequency and hours were 4.7 ± 2.1 times/week and 7.8 ± 6.7 hours/week, respectively. In total, 24 kinds of exercise were reported, and 169 (69.8%) patients noted more than one type of exercise. Among the 24 kinds of primary exercises, the most common exercise was active walking (*n* = 129, 49.0%), followed by stretching (Table 2). When we classified all the exercises into superior categories (i.e., aerobic, resistance, and stretching), aerobic exercise was the most common (71.9%). Additionally, 78.7% of the participants exercised at home, and solo exercise was reported by more than 90% (91.5%). In terms of exercise intensity, exercise with a mild intensity was the most common (45.6%), followed by moderate intensity exercise.

### 3.3. Comparison of Exercise Patterns between Mild and Advanced PD Patients

When we compared exercise patterns between the two groups, the amount of exercise was significantly lower in the advanced PD group than in the mild PD group, while there was no difference in the frequency of exercise (Table 1). Regarding the exercise intensity, moderate-to-high intensity exercise was performed by 57.6% of the mild PD participants, but only 28.3% of the moderate-to-severe PD patients. While a wide variety of exercises (a total of 24 kinds) was reported by the mild PD group, only 8 types of exercises were reported in the advanced PD group (Table 2). Active walking was the most frequently reported primary exercise in both groups (50% and 45.2% in the mild and advanced PD groups, respectively). There was no difference in the category of exercise between the two groups, but more patients in the advanced group did home-based exercise and solo exercise compared to the mild PD group.

### 3.4. Physical Activity Level of PD Patients

The mean K-PASE score of all PD patients was 51.52 ± 45.36, and the mean subscores for each section were 25.7 ± 22.1, 6.8 ± 25.9, and 19.0 ± 21.4 for leisure exercise, work or volunteering, and household chores, respectively (Table 1). When we compared the mean PASE scores between the two groups, the advanced PD group showed a significantly lower total PASE score (*p* < 0.001) and leisure exercise subscore (*p* < 0.001) than the mild PD group. There was no significant difference in PASE work/volunteering and household chores between the two groups.

## 4. Discussion

To our knowledge, this is the first study to examine the detailed daily exercise patterns and physical activity levels of PD patients in the mild to advanced stages. The advanced PD group reported a lower amount and intensity of exercise and less physical activity compared to the mild PD group. Considering there was no significant difference in the frequency of exercise, advanced PD patients tended to spend less time completing each type of exercise. Based on our results, the exercise recommendations for advanced PD patients should focus on how to increase the duration of each session of exercise and to increase the exercise intensity due to limited mobility.

Not only the decrement of amount and intensity in exercise but also the loss of diversity of exercise was observed in the advanced group. Only 8 kinds of exercise were done in the advanced PD group, whereas 24 types were reported by patients in the mild PD group. Exercises requiring a higher level of balance, like ball games, yoga, or mountain hiking, were not reported in the advanced PD group.

Still, it is encouraging that both mild and advanced PD patients in this study were exercising properly with regards to the duration and frequency of exercise that is recommended by current guidelines [7, 8]. PD patients did exercise regularly (4.7 times a week on average), and the most common exercise pattern was aerobic exercise with a mild-to-moderate intensity. Although there has been no head-to-head comparison study between elderly controls and PD patients, PD patients exercise as much as healthy Korean elderly individuals based on the K-PASE score of healthy Korean elderly people from a previous study [24]. There was no significant difference (according to Mann–Whitney *U*-tests) between the mean PASE leisure exercise score of PD patients (25.7 ± 22.0) and that of healthy Korean elderly individuals (24.6 ± 24.6).

However, the household chores or working scores of PD patients were significantly lower than those of healthy elderly individuals (*p*=0.008). In addition, compared to the PASE scores of PD patients from the United States (US) [6, 25], our South Korean PD patients did substantially fewer household chores and work/volunteering activities, making their total PASE scores lower. This finding implies that exercise contributes to a large portion of physical activity compared to household chores or working in Korean PD patients.

The most frequently performed exercise in both groups was active walking, which is easily accessible and does not require any equipment or specific places. It is difficult to directly compare our results with PD patients in other countries due to the lack of similar studies, but active walking is a popular exercise not only in PD patients but in the general population in South Korea. Accordingly, a previous study revealed the mean number of daily steps was 5,755 (8^th^) in South Korea, while the US ranked 29^th^ with 4,774 steps out of 46 countries [26]. Since PD can present with various gait symptoms, active walking is often regarded as an effective exercise for PD patients. However, combined exercises from various categories are typically recommended for PD patients [7, 8]. Therefore, it is also important to recommend that PD patients combine other resistance exercises or stretching for flexibility with walking.

Our study showed most PD patients performed solo exercise, and this tendency was stronger in advanced PD patients. Group exercise offers social support and bonding with other patients, which is important to motivate PD patients to continue exercising. Additionally, most PD patients did home-based exercise, which indicates that it is not easy for PD patients to access sports facilities and perform sophisticated exercises [27]. Sports facilities are equipped with exercise coaches and various exercising equipment, which allow people to take part in exercise of a higher intensity. Therefore, these higher intensity exercises should be recommended to patients in the mild PD group who already took part in a fair amount of low-intensity exercise.

Our study has strong points. We not only cataloged information about the duration, frequency, and specific type of exercise done by mild to advanced PD patients in their daily lives but also objectively supported and quantified those exercise patterns using the PASE scores. Still this study has some limitations. First, while investigating the exercise patterns of PD patients, we relied on subjective patient reports, which may have introduced reporting and recall biases. However, to address this potential limitation, we used PASE, a validated measurement tool of physical activity that has been demonstrated to correlate with objective measures of aerobic capacity [23]. The PASE was used in a previous study to evaluate physical activity in patients with early PD [6, 25]. Second, the presence of nonmotor symptoms, which may affect the quality and amount of exercise performed, was not investigated. Instead, while enrolling patients, we excluded people who reported dementia or psychiatric problems that could have significantly influenced their exercise patterns. At the same time, however, it is possible that the amount of exercise and physical activity of the advanced PD patients might have been overestimated. Third, we did not enroll a normal matched elderly population to use as a control group and compare with the PD participants. However, a report of the physical activity of a normal elderly population in South Korea using the same scale (K-PASE) has already been published; thus, we were able to use those results indirectly. Last, this was a cross-sectional observational study, so it was difficult to investigate direct changes associated with disease progression and also to show the prognosis based on the exercise level alone in PD patients.

In conclusion, our study demonstrated the real-life exercise patterns of PD patients and the differences between mild and advanced PD patients, which can serve as baseline data for clinicians to plan well-organized, practical exercise regimens for PD patients. The exercise activities reported by PD patients mainly included aerobic exercise with a mild-to-moderate intensity. Based on our results, a greater emphasis on a higher intensity of exercise is needed in the mild PD group, while the advanced PD group requires an increasing amount of time for each exercise session. Future studies will be needed to determine more personalized exercise recommendations for these PD patients.

## Figures and Tables

**Table 1:** Demographics, exercise patterns, and PASE scores of Parkinson's disease patients.

	Total (*n* = 263)	HY 1-2 (*n* = 210)	HY 3-4 (*n* = 53)	*P* value
Demographics				
Age (years)	67.7 ± 29.2	66.5 ± 9.2	72.3 ± 7.1	<0.001
Male, *n* (%)	129 (49.0)	104 (49.5)	25 (47.2)	0.759
BMI (kg/m^2^)	24.8 ± 5.8	24.6 ± 3.3	25.5 ± 11.2	0.276
Disease duration (years)	8.3 ± 5.2	7.4 ± 4.6	11.5 ± 6.1	<0.001
UPDRS part 3	17.6 ± 8.2	15.9 ± 8.2	24.1 ± 8.6	<0.001
LEDD (mg/day)	610.8 ± 400.4	535.9 ± 354.2	907.6 ± 437.0	<0.001
Exercise behaviors				
Frequency (times/week)	4.7 ± 2.1	4.8 ± 2.1	4.3 ± 2.2	0.281
Exercise amount (hours/week)	7.8 ± 6.7	8.5 ± 6.9	5.0 ± 4.9	<0.001
Exercise intensity (mild/moderate/high), *n* (%)	120 (45.6)/93 (35.4)/43 (16.3)	88 (41.9)/82 (39.0)/39 (18.6)	32 (60.4)/11 (20.8)/4 (7.5)	<0.001
Categories of primary exercise, *n* (%)				
None	7 (2.7)	1 (0.5)	6 (11.3)	—
Aerobic/resistance/stretching	189 (71.9)/43 (16.3)/24 (9.1)	152 (72.4)/39 (18.6)/18 (8.6)	37 (69.8)/4 (7.5)/6 (11.3)	—
Home-based/ sports facility-based	207 (78.7)/49 (18.6)	164 (78.1)/45 (21.4)	43 (81.1)/4 (7.5)	0.020
Group exercise	22 (8.4)	22 (10.5)	0	0.010
PASE (total score)	51.5 ± 45.4	55.9 ± 47.1	34.1 ± 32.8	<0.001
Leisure exercise	25.7 ± 22.0	28.2 ± 22.7	16.2 ± 16.2	<0.001
Work/volunteering	6.8 ± 25.9	7.5 ± 27.1	4.0 ± 20.2	0.368
Household	19.0 ± 21.4	20.3 ± 22.3	13.9 ± 17.0	0.092

HY, Hoehn–Yahr stage; BMI, body mass index; UPDRS, unified Parkinson's disease rating sale; LEDD, levodopa equivalent daily dose; PASE, Physical Activity Scale of the Elderly.

**Table 2:** Rank of the specific exercises reported by patients.

Rank	Specific exercises			
	Total	Patients	HY 3-4	Patients
1	Active walking	129	Active walking	32
2	Stretching	21	Stretching	5
3	Table tennis	12	Climbing stairs	3
4	Push-ups, squatting, stationary bike, working out at fitness clubs	10	Stationary bike, squatting	2
6	Yoga	9	Physical therapy, weight training, yoga	1
7	Climbing stairs	6		
8	Outside biking, running	5		
9	Climbing mountains, golf, swimming	4		
10	Badminton, lifting dumbbells, Tai Chi, water aerobics	2		
Else	Basketball, billiards, bowling, dancing (ball room, aerobics), football, physical therapy, Pilates, pull-ups	1		

## Data Availability

All data generated or analysed during this study are included in this published article (and its supplementary information files).

## Supplementary Materials

Supplementary Materials Supplementary Table 1. Specific exercises reported by patients and their exercise categories.
