# Supplementary material for: Daily Exercise Patterns and Their Differences between Parkinson's Disease Patients with and without Postural Instability
Source: Parkinsons Dis. 2022 May 19;2022:3191598. doi: 10.1155/2022/3191598 (PMC9135569; doi:10.1155/2022/3191598)
Supplement: Supplementary Materials — Supplementary Table 1. Specific exercises reported by patients and their exercise categories. [file 3191598.f1.docx]

Supplementary Table 1. Specific exercises reported by patients and their exercise categories

| **Exercise categories** | **Specific exercises** |
| --- | --- |
| **Categories according to physiology of exercise** | |
| Aerobic exercise | Active walking, badminton, basketball, billiard, biking, bowling, climbing (stairs or mountains), dancing, football, golf, running, swimming, table tennis, water aerobics |
| Resistance exercise | Lifting dumbbells, Pilates, pull-ups, push-ups, squatting, Yoga Working out at fitness clubs (weight training) |
| Stretching (flexibility) | Stretching, physical therapy, Tai-Chi |
| **Other categories** | |
| Exercise at sports facilities | Ballroom dancing, golf, physical therapy, Pilates, table tennis, swimming, water aerobics, working out at fitness clubs, Yoga |
| Group exercise | Badminton, basketball, billiards, dancing, football, table tennis |
